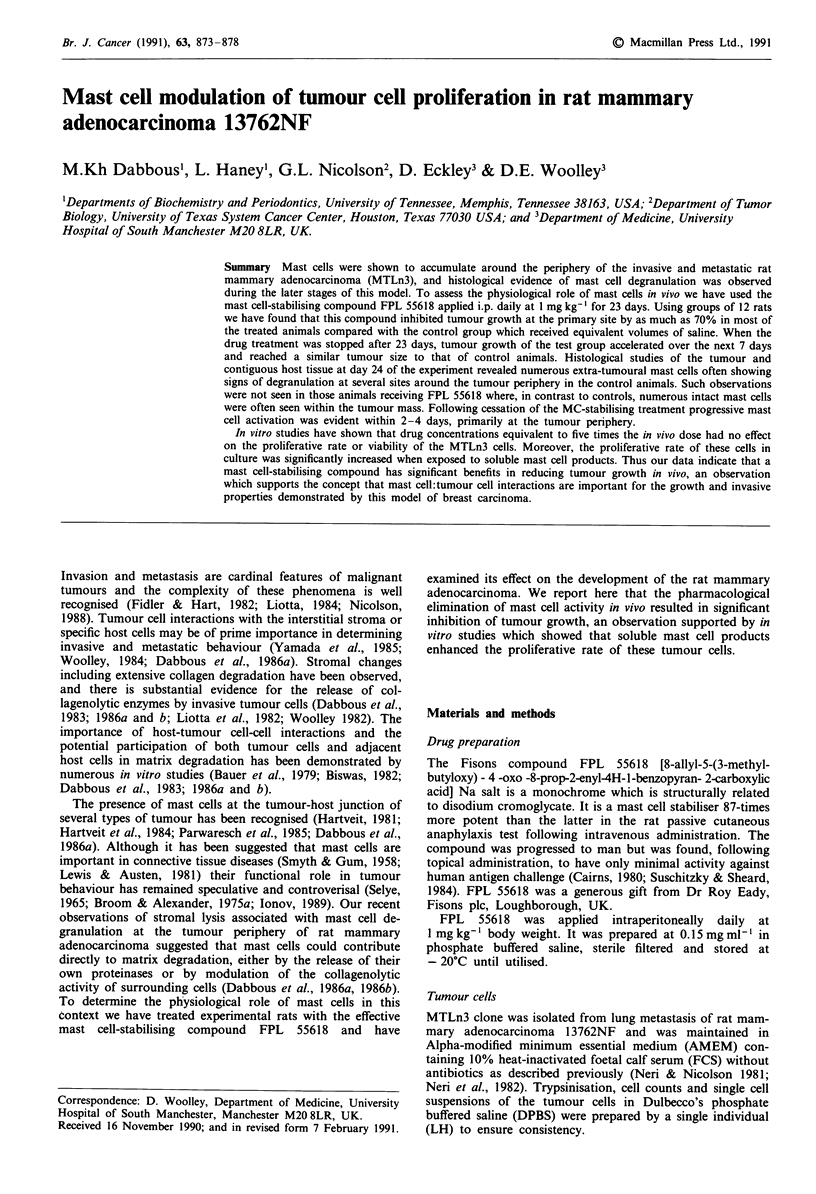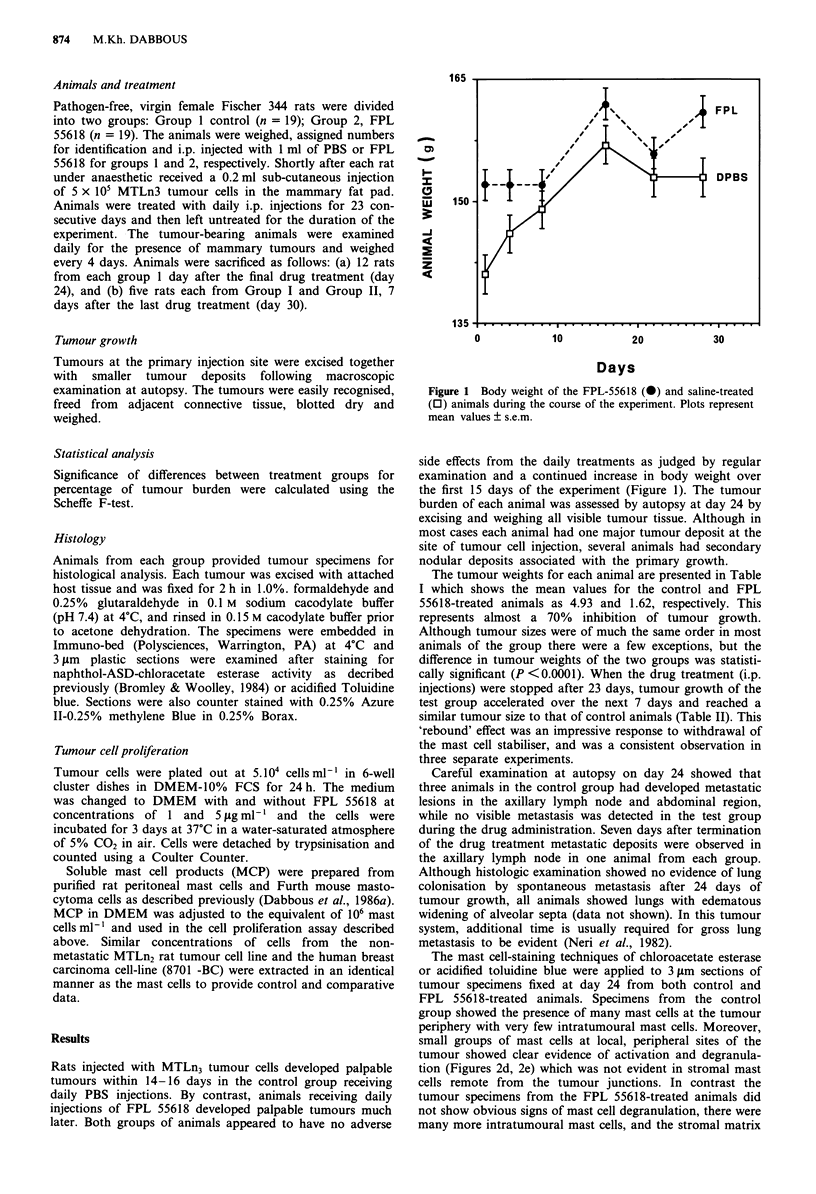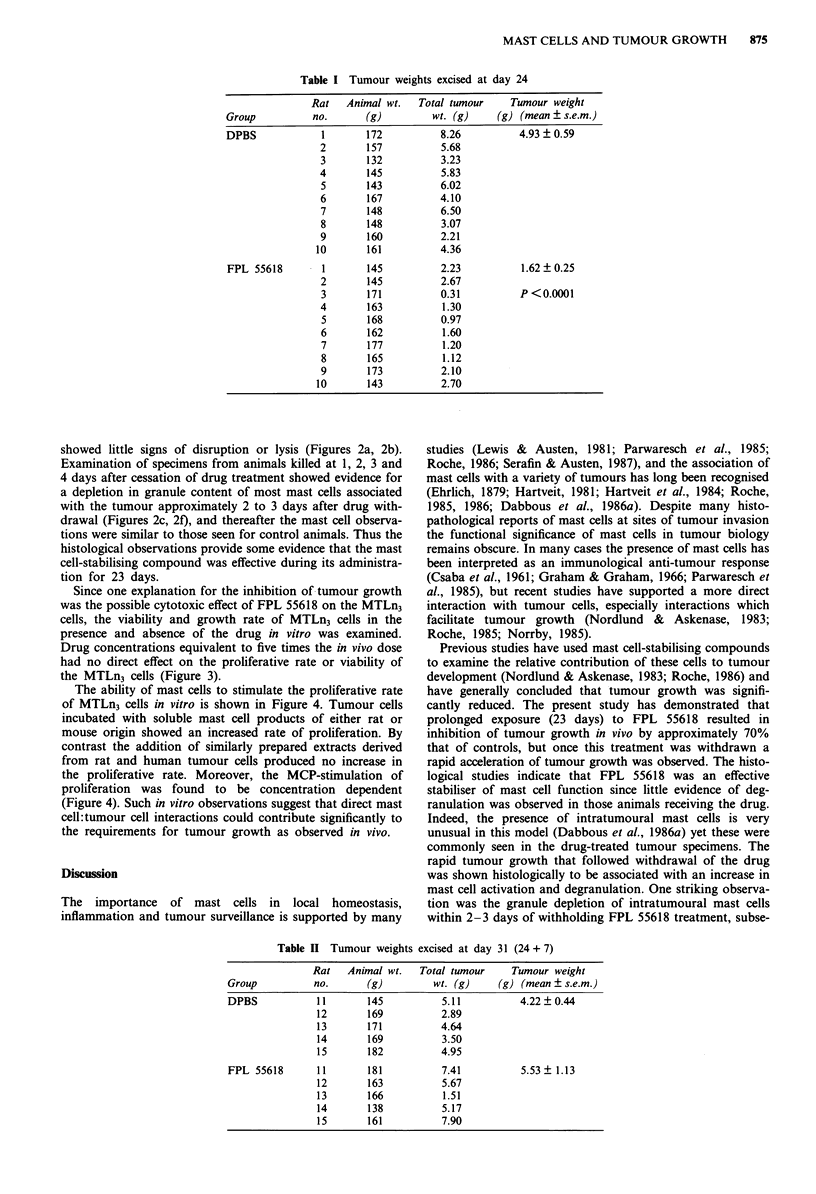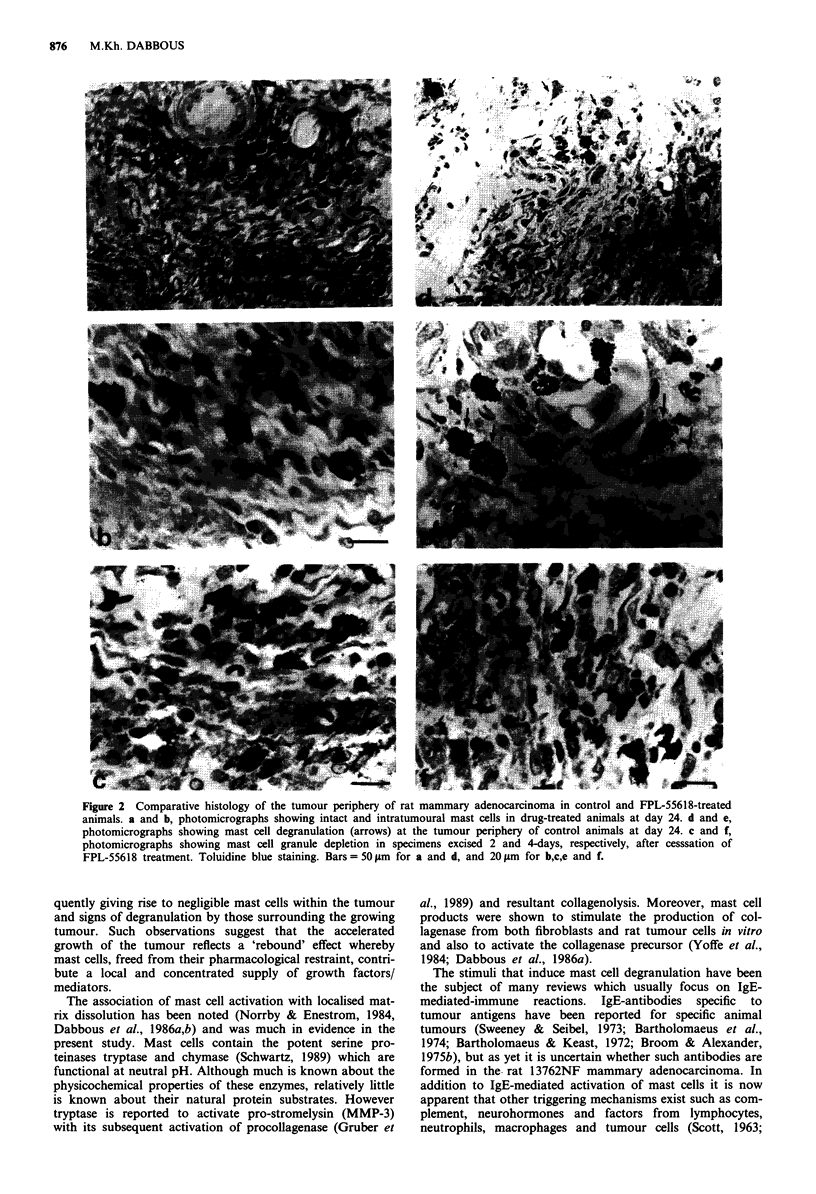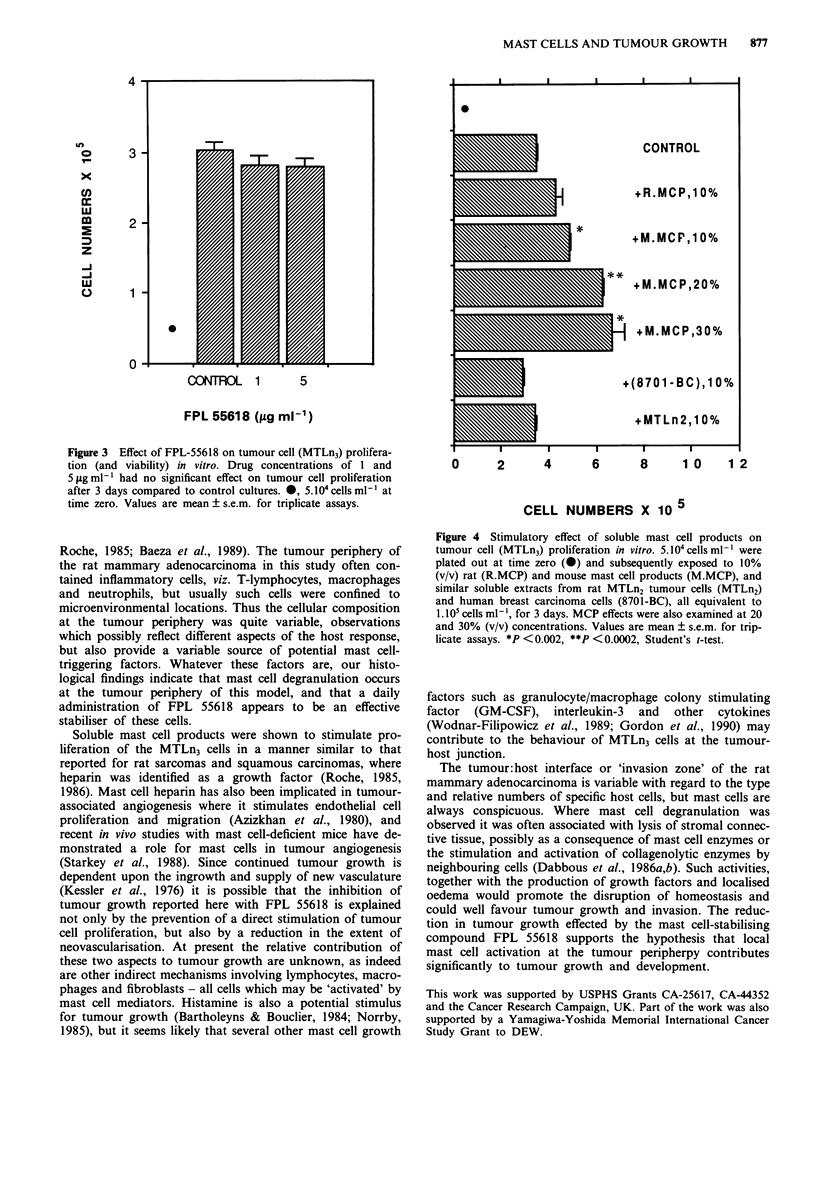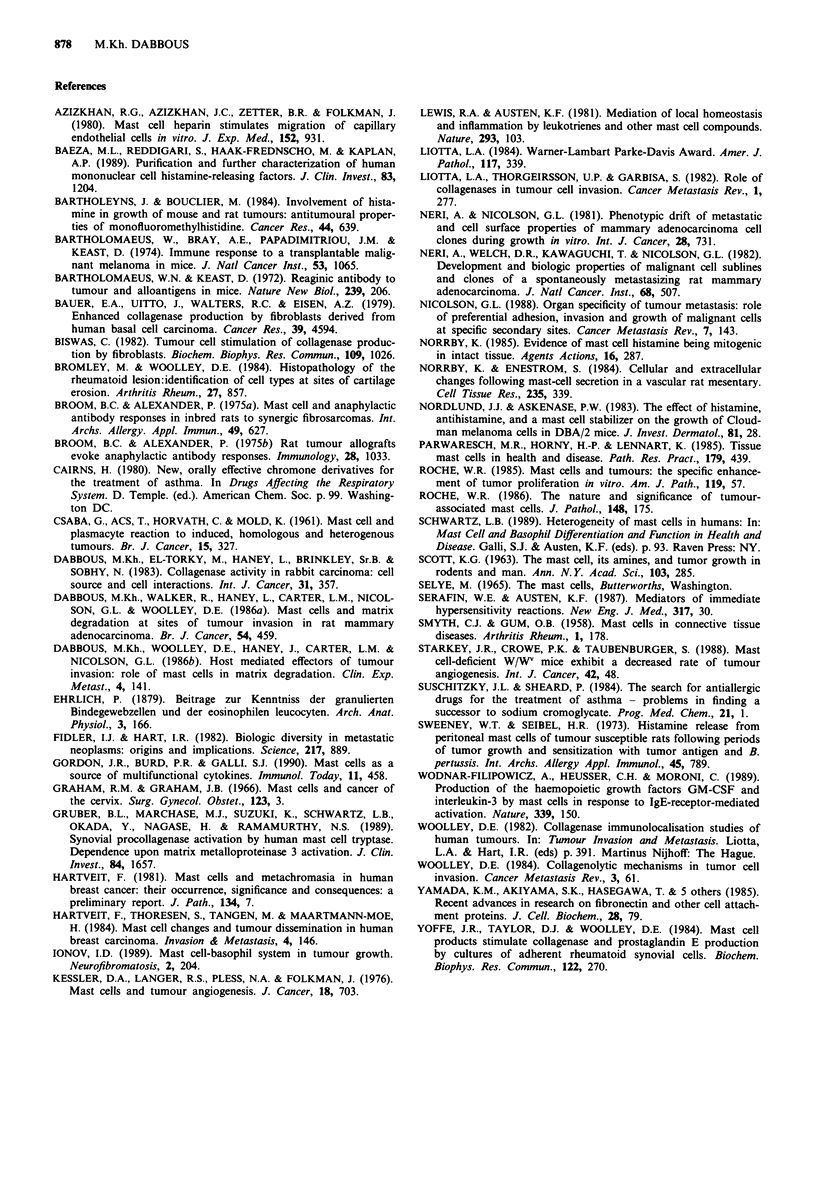# Mast cell modulation of tumour cell proliferation in rat mammary adenocarcinoma 13762NF.

**DOI:** 10.1038/bjc.1991.192

**Published:** 1991-06

**Authors:** M. K. Dabbous, L. Haney, G. L. Nicolson, D. Eckley, D. E. Woolley

**Affiliations:** Department of Biochemistry, University of Tennessee, Memphis 38163.

## Abstract

**Images:**


Br. J. Cancer (1991), 63, 873-878 © Macmillan Press Ltd., 1991

### Mast cell modulation of tumour cell proliferation in rat mammary adenocarcinoma 13762NF

M.Kh Dabbous[1], L. Haney[1], G.L. Nicolson[2], D. Eckley[3] & D.E. Woolley[3]

[1]*Departments of Biochemistry and Periodontics, University of Tennessee, Memphis, Tennessee 38163, USA;* [2]*Department of Tumor Biology, University of Texas System Cancer Center, Houston, Texas 77030 USA; and* [3]*Department of Medicine, University Hospital of South Manchester M20 8LR, UK.*

**Summary** Mast cells were shown to accumulate around the periphery of the invasive and metastatic rat mammary adenocarcinoma (MTLn3), and histological evidence of mast cell degranulation was observed during the later stages of this model. To assess the physiological role of mast cells *in vivo* we have used the mast cell-stabilising compound FPL 55618 applied i.p. daily at 1 mg $kg^{-1}$ for 23 days. Using groups of 12 rats we have found that this compound inhibited tumour growth at the primary site by as much as 70% in most of the treated animals compared with the control group which received equivalent volumes of saline. When the drug treatment was stopped after 23 days, tumour growth of the test group accelerated over the next 7 days and reached a similar tumour size to that of control animals. Histological studies of the tumour and contiguous host tissue at day 24 of the experiment revealed numerous extra-tumoural mast cells often showing signs of degranulation at several sites around the tumour periphery in the control animals. Such observations were not seen in those animals receiving FPL 55618 where, in contrast to controls, numerous intact mast cells were often seen within the tumour mass. Following cessation of the MC-stabilising treatment progressive mast cell activation was evident within 2-4 days, primarily at the tumour periphery.

*In vitro* studies have shown that drug concentrations equivalent to five times the *in vivo* dose had no effect on the proliferative rate or viability of the MTLn3 cells. Moreover, the proliferative rate of these cells in culture was significantly increased when exposed to soluble mast cell products. Thus our data indicate that a mast cell-stabilising compound has significant benefits in reducing tumour growth *in vivo*, an observation which supports the concept that mast cell:tumour cell interactions are important for the growth and invasive properties demonstrated by this model of breast carcinoma.

Invasion and metastasis are cardinal features of malignant tumours and the complexity of these phenomena is well recognised (Fidler & Hart, 1982; Liotta, 1984; Nicolson, 1988). Tumour cell interactions with the interstitial stroma or specific host cells may be of prime importance in determining invasive and metastatic behaviour (Yamada *et al.*, 1985; Woolley, 1984; Dabbous *et al.*, 1986*a*). Stromal changes including extensive collagen degradation have been observed, and there is substantial evidence for the release of collagenolytic enzymes by invasive tumour cells (Dabbous *et al.*, 1983; 1986*a* and *b*; Liotta *et al.*, 1982; Woolley 1982). The importance of host-tumour cell-cell interactions and the potential participation of both tumour cells and adjacent host cells in matrix degradation has been demonstrated by numerous *in vitro* studies (Bauer *et al.*, 1979; Biswas, 1982; Dabbous *et al.*, 1983; 1986*a* and *b*).

The presence of mast cells at the tumour-host junction of several types of tumour has been recognised (Hartveit, 1981; Hartveit *et al.*, 1984; Parwaresch *et al.*, 1985; Dabbous *et al.*, 1986*a*). Although it has been suggested that mast cells are important in connective tissue diseases (Smyth & Gum, 1958; Lewis & Austen, 1981) their functional role in tumour behaviour has remained speculative and controverisal (Selye, 1965; Broom & Alexander, 1975*a*; Ionov, 1989). Our recent observations of stromal lysis associated with mast cell degranulation at the tumour periphery of rat mammary adenocarcinoma suggested that mast cells could contribute directly to matrix degradation, either by the release of their own proteinases or by modulation of the collagenolytic activity of surrounding cells (Dabbous *et al.*, 1986*a*, 1986*b*). To determine the physiological role of mast cells in this context we have treated experimental rats with the effective mast cell-stabilising compound FPL 55618 and have examined its effect on the development of the rat mammary adenocarcinoma. We report here that the pharmacological elimination of mast cell activity *in vivo* resulted in significant inhibition of tumour growth, an observation supported by *in vitro* studies which showed that soluble mast cell products enhanced the proliferative rate of these tumour cells.

Correspondence: D. Woolley, Department of Medicine, University Hospital of South Manchester, Manchester M20 8LR, UK.
Received 16 November 1990; and in revised form 7 February 1991.

### Materials and methods

### *Drug preparation*

The Fisons compound FPL 55618 [8-allyl-5-(3-methylbutyloxy) - 4 -oxo -8-prop-2-enyl-4H-1-benzopyran- 2-carboxylic acid] Na salt is a monochrome which is structurally related to disodium cromoglycate. It is a mast cell stabiliser 87-times more potent than the latter in the rat passive cutaneous anaphylaxis test following intravenous administration. The compound was progressed to man but was found, following topical administration, to have only minimal activity against human antigen challenge (Cairns, 1980; Suschitzky & Sheard, 1984). FPL 55618 was a generous gift from Dr Roy Eady, Fisons plc, Loughborough, UK.

FPL 55618 was applied intraperitoneally daily at 1 mg $kg^{-1}$ body weight. It was prepared at 0.15 mg $ml^{-1}$ in phosphate buffered saline, sterile filtered and stored at $-20°C$ until utilised.

### *Tumour cells*

MTLn3 clone was isolated from lung metastasis of rat mammary adenocarcinoma 13762NF and was maintained in Alpha-modified minimum essential medium (AMEM) containing 10% heat-inactivated foetal calf serum (FCS) without antibiotics as described previously (Neri & Nicolson 1981; Neri *et al.*, 1982). Trypsinisation, cell counts and single cell suspensions of the tumour cells in Dulbecco's phosphate buffered saline (DPBS) were prepared by a single individual (LH) to ensure consistency.

*Animals and treatment*

Pathogen-free, virgin female Fischer 344 rats were divided into two groups: Group 1 control ($n$ = 19); Group 2, FPL 55618 ($n$ = 19). The animals were weighed, assigned numbers for identification and i.p. injected with 1 ml of PBS or FPL 55618 for groups 1 and 2, respectively. Shortly after each rat under anaesthetic received a 0.2 ml sub-cutaneous injection of $5 \times 10^5$ MTLn3 tumour cells in the mammary fat pad. Animals were treated with daily i.p. injections for 23 consecutive days and then left untreated for the duration of the experiment. The tumour-bearing animals were examined daily for the presence of mammary tumours and weighed every 4 days. Animals were sacrificed as follows: (a) 12 rats from each group 1 day after the final drug treatment (day 24), and (b) five rats each from Group I and Group II, 7 days after the last drug treatment (day 30).

*Tumour growth*

Tumours at the primary injection site were excised together with smaller tumour deposits following macroscopic examination at autopsy. The tumours were easily recognised, freed from adjacent connective tissue, blotted dry and weighed.

*Statistical analysis*

Significance of differences between treatment groups for percentage of tumour burden were calculated using the Scheffe F-test.

*Histology*

Animals from each group provided tumour specimens for histological analysis. Each tumour was excised with attached host tissue and was fixed for 2 h in 1.0%. formaldehyde and 0.25% glutaraldehyde in 0.1 M sodium cacodylate buffer (pH 7.4) at 4°C, and rinsed in 0.15 M cacodylate buffer prior to acetone dehydration. The specimens were embedded in Immuno-bed (Polysciences, Warrington, PA) at 4°C and 3 μm plastic sections were examined after staining for naphthol-ASD-chloracetate esterase activity as decribed previously (Bromley & Woolley, 1984) or acidified Toluidine blue. Sections were also counter stained with 0.25% Azure II-0.25% methylene Blue in 0.25% Borax.

*Tumour cell proliferation*

Tumour cells were plated out at 5.10$^4$ cells ml$^{-1}$ in 6-well cluster dishes in DMEM-10% FCS for 24 h. The medium was changed to DMEM with and without FPL 55618 at concentrations of 1 and 5 μg ml$^{-1}$ and the cells were incubated for 3 days at 37°C in a water-saturated atmosphere of 5% $CO_2$ in air. Cells were detached by trypsinisation and counted using a Coulter Counter.

Soluble mast cell products (MCP) were prepared from purified rat peritoneal mast cells and Furth mouse mastocytoma cells as described previously (Dabbous *et al.*, 1986*a*). MCP in DMEM was adjusted to the equivalent of $10^6$ mast cells ml$^{-1}$ and used in the cell proliferation assay described above. Similar concentrations of cells from the non-metastatic $MTLn_2$ rat tumour cell line and the human breast carcinoma cell-line (8701 -BC) were extracted in an identical manner as the mast cells to provide control and comparative data.

**Results**

Rats injected with $MTLn_3$ tumour cells developed palpable tumours within 14–16 days in the control group receiving daily PBS injections. By contrast, animals receiving daily injections of FPL 55618 developed palpable tumours much later. Both groups of animals appeared to have no adverse side effects from the daily treatments as judged by regular examination and a continued increase in body weight over the first 15 days of the experiment (Figure 1). The tumour burden of each animal was assessed by autopsy at day 24 by excising and weighing all visible tumour tissue. Although in most cases each animal had one major tumour deposit at the site of tumour cell injection, several animals had secondary nodular deposits associated with the primary growth.

**Figure 1** Body weight of the FPL-55618 (●) and saline-treated (□) animals during the course of the experiment. Plots represent mean values ± s.e.m.

The tumour weights for each animal are presented in Table I which shows the mean values for the control and FPL 55618-treated animals as 4.93 and 1.62, respectively. This represents almost a 70% inhibition of tumour growth. Although tumour sizes were of much the same order in most animals of the group there were a few exceptions, but the difference in tumour weights of the two groups was statistically significant ($P < 0.0001$). When the drug treatment (i.p. injections) were stopped after 23 days, tumour growth of the test group accelerated over the next 7 days and reached a similar tumour size to that of control animals (Table II). This 'rebound' effect was an impressive response to withdrawal of the mast cell stabiliser, and was a consistent observation in three separate experiments.

Careful examination at autopsy on day 24 showed that three animals in the control group had developed metastatic lesions in the axillary lymph node and abdominal region, while no visible metastasis was detected in the test group during the drug administration. Seven days after termination of the drug treatment metastatic deposits were observed in the axillary lymph node in one animal from each group. Although histologic examination showed no evidence of lung colonisation by spontaneous metastasis after 24 days of tumour growth, all animals showed lungs with edematous widening of alveolar septa (data not shown). In this tumour system, additional time is usually required for gross lung metastasis to be evident (Neri *et al.*, 1982).

The mast cell-staining techniques of chloroacetate esterase or acidified toluidine blue were applied to 3 μm sections of tumour specimens fixed at day 24 from both control and FPL 55618-treated animals. Specimens from the control group showed the presence of many mast cells at the tumour periphery with very few intratumoural mast cells. Moreover, small groups of mast cells at local, peripheral sites of the tumour showed clear evidence of activation and degranulation (Figures 2d, 2e) which was not evident in stromal mast cells remote from the tumour junctions. In contrast the tumour specimens from the FPL 55618-treated animals did not show obvious signs of mast cell degranulation, there were many more intratumoural mast cells, and the stromal matrix

**Table I** Tumour weights excised at day 24

| Group | Rat no. | Animal wt. (g) | Total tumour wt. (g) | Tumour weight (g) (mean ± s.e.m.) |
|---|---|---|---|---|
| DPBS | 1 | 172 | 8.26 | 4.93 ± 0.59 |
| | 2 | 157 | 5.68 | |
| | 3 | 132 | 3.23 | |
| | 4 | 145 | 5.83 | |
| | 5 | 143 | 6.02 | |
| | 6 | 167 | 4.10 | |
| | 7 | 148 | 6.50 | |
| | 8 | 148 | 3.07 | |
| | 9 | 160 | 2.21 | |
| | 10 | 161 | 4.36 | |
| FPL 55618 | 1 | 145 | 2.23 | 1.62 ± 0.25 |
| | 2 | 145 | 2.67 | |
| | 3 | 171 | 0.31 | $P < 0.0001$ |
| | 4 | 163 | 1.30 | |
| | 5 | 168 | 0.97 | |
| | 6 | 162 | 1.60 | |
| | 7 | 177 | 1.20 | |
| | 8 | 165 | 1.12 | |
| | 9 | 173 | 2.10 | |
| | 10 | 143 | 2.70 | |

showed little signs of disruption or lysis (Figures 2a, 2b). Examination of specimens from animals killed at 1, 2, 3 and 4 days after cessation of drug treatment showed evidence for a depletion in granule content of most mast cells associated with the tumour approximately 2 to 3 days after drug withdrawal (Figures 2c, 2f), and thereafter the mast cell observations were similar to those seen for control animals. Thus the histological observations provide some evidence that the mast cell-stabilising compound was effective during its administration for 23 days.

Since one explanation for the inhibition of tumour growth was the possible cytotoxic effect of FPL 55618 on the $MTLn_3$ cells, the viability and growth rate of $MTLn_3$ cells in the presence and absence of the drug *in vitro* was examined. Drug concentrations equivalent to five times the *in vivo* dose had no direct effect on the proliferative rate or viability of the $MTLn_3$ cells (Figure 3).

The ability of mast cells to stimulate the proliferative rate of $MTLn_3$ cells *in vitro* is shown in Figure 4. Tumour cells incubated with soluble mast cell products of either rat or mouse origin showed an increased rate of proliferation. By contrast the addition of similarly prepared extracts derived from rat and human tumour cells produced no increase in the proliferative rate. Moreover, the MCP-stimulation of proliferation was found to be concentration dependent (Figure 4). Such *in vitro* observations suggest that direct mast cell:tumour cell interactions could contribute significantly to the requirements for tumour growth as observed *in vivo*.

### Discussion

The importance of mast cells in local homeostasis, inflammation and tumour surveillance is supported by many studies (Lewis & Austen, 1981; Parwaresch *et al.*, 1985; Roche, 1986; Serafin & Austen, 1987), and the association of mast cells with a variety of tumours has long been recognised (Ehrlich, 1879; Hartveit, 1981; Hartveit *et al.*, 1984; Roche, 1985, 1986; Dabbous *et al.*, 1986*a*). Despite many histopathological reports of mast cells at sites of tumour invasion the functional significance of mast cells in tumour biology remains obscure. In many cases the presence of mast cells has been interpreted as an immunological anti-tumour response (Csaba *et al.*, 1961; Graham & Graham, 1966; Parwaresch *et al.*, 1985), but recent studies have supported a more direct interaction with tumour cells, especially interactions which facilitate tumour growth (Nordlund & Askenase, 1983; Roche, 1985; Norrby, 1985).

Previous studies have used mast cell-stabilising compounds to examine the relative contribution of these cells to tumour development (Nordlund & Askenase, 1983; Roche, 1986) and have generally concluded that tumour growth was significantly reduced. The present study has demonstrated that prolonged exposure (23 days) to FPL 55618 resulted in inhibition of tumour growth *in vivo* by approximately 70% that of controls, but once this treatment was withdrawn a rapid acceleration of tumour growth was observed. The histological studies indicate that FPL 55618 was an effective stabiliser of mast cell function since little evidence of degranulation was observed in those animals receiving the drug. Indeed, the presence of intratumoural mast cells is very unusual in this model (Dabbous *et al.*, 1986*a*) yet these were commonly seen in the drug-treated tumour specimens. The rapid tumour growth that followed withdrawal of the drug was shown histologically to be associated with an increase in mast cell activation and degranulation. One striking observation was the granule depletion of intratumoural mast cells within 2–3 days of withholding FPL 55618 treatment, subse-

**Table II** Tumour weights excised at day 31 (24 + 7)

| Group | Rat no. | Animal wt. (g) | Total tumour wt. (g) | Tumour weight (g) (mean ± s.e.m.) |
|---|---|---|---|---|
| DPBS | 11 | 145 | 5.11 | 4.22 ± 0.44 |
| | 12 | 169 | 2.89 | |
| | 13 | 171 | 4.64 | |
| | 14 | 169 | 3.50 | |
| | 15 | 182 | 4.95 | |
| FPL 55618 | 11 | 181 | 7.41 | 5.53 ± 1.13 |
| | 12 | 163 | 5.67 | |
| | 13 | 166 | 1.51 | |
| | 14 | 138 | 5.17 | |
| | 15 | 161 | 7.90 | |

**Figure 2** Comparative histology of the tumour periphery of rat mammary adenocarcinoma in control and FPL-55618-treated animals. **a** and **b**, photomicrographs showing intact and intratumoural mast cells in drug-treated animals at day 24. **d** and **e**, photomicrographs showing mast cell degranulation (arrows) at the tumour periphery of control animals at day 24. **c** and **f**, photomicrographs showing mast cell granule depletion in specimens excised 2 and 4-days, respectively, after cesssation of FPL-55618 treatment. Toluidine blue staining. Bars = 50 μm for **a** and **d**, and 20 μm for **b**,**c**,**e** and **f**.

quently giving rise to negligible mast cells within the tumour and signs of degranulation by those surrounding the growing tumour. Such observations suggest that the accelerated growth of the tumour reflects a 'rebound' effect whereby mast cells, freed from their pharmacological restraint, contribute a local and concentrated supply of growth factors/mediators.

The association of mast cell activation with localised matrix dissolution has been noted (Norrby & Enestrom, 1984, Dabbous *et al.*, 1986*a*,*b*) and was much in evidence in the present study. Mast cells contain the potent serine proteinases tryptase and chymase (Schwartz, 1989) which are functional at neutral pH. Although much is known about the physicochemical properties of these enzymes, relatively little is known about their natural protein substrates. However tryptase is reported to activate pro-stromelysin (MMP-3) with its subsequent activation of procollagenase (Gruber *et al.*, 1989) and resultant collagenolysis. Moreover, mast cell products were shown to stimulate the production of collagenase from both fibroblasts and rat tumour cells *in vitro* and also to activate the collagenase precursor (Yoffe *et al.*, 1984; Dabbous *et al.*, 1986*a*).

The stimuli that induce mast cell degranulation have been the subject of many reviews which usually focus on IgE-mediated-immune reactions. IgE-antibodies specific to tumour antigens have been reported for specific animal tumours (Sweeney & Seibel, 1973; Bartholomaeus *et al.*, 1974; Bartholomaeus & Keast, 1972; Broom & Alexander, 1975*b*), but as yet it is uncertain whether such antibodies are formed in the rat 13762NF mammary adenocarcinoma. In addition to IgE-mediated activation of mast cells it is now apparent that other triggering mechanisms exist such as complement, neurohormones and factors from lymphocytes, neutrophils, macrophages and tumour cells (Scott, 1963;

**Figure 3** Effect of FPL-55618 on tumour cell ($MTLn_3$) proliferation (and viability) *in vitro*. Drug concentrations of 1 and 5 $\mu g\ ml^{-1}$ had no significant effect on tumour cell proliferation after 3 days compared to control cultures. ●, $5.10^4$ cells $ml^{-1}$ at time zero. Values are mean ± s.e.m. for triplicate assays.

**Figure 4** Stimulatory effect of soluble mast cell products on tumour cell ($MTLn_3$) proliferation *in vitro*. $5.10^4$ cells $ml^{-1}$ were plated out at time zero (●) and subsequently exposed to 10% (v/v) rat (R.MCP) and mouse mast cell products (M.MCP), and similar soluble extracts from rat $MTLn_2$ tumour cells ($MTLn_2$) and human breast carcinoma cells (8701-BC), all equivalent to $1.10^5$ cells $ml^{-1}$, for 3 days. MCP effects were also examined at 20 and 30% (v/v) concentrations. Values are mean ± s.e.m. for triplicate assays. *$P<0.002$, **$P<0.0002$, Student's *t*-test.

Roche, 1985; Baeza *et al.*, 1989). The tumour periphery of the rat mammary adenocarcinoma in this study often contained inflammatory cells, *viz.* T-lymphocytes, macrophages and neutrophils, but usually such cells were confined to microenvironmental locations. Thus the cellular composition at the tumour periphery was quite variable, observations which possibly reflect different aspects of the host response, but also provide a variable source of potential mast cell-triggering factors. Whatever these factors are, our histological findings indicate that mast cell degranulation occurs at the tumour periphery of this model, and that a daily administration of FPL 55618 appears to be an effective stabiliser of these cells.

Soluble mast cell products were shown to stimulate proliferation of the $MTLn_3$ cells in a manner similar to that reported for rat sarcomas and squamous carcinomas, where heparin was identified as a growth factor (Roche, 1985, 1986). Mast cell heparin has also been implicated in tumour-associated angiogenesis where it stimulates endothelial cell proliferation and migration (Azizkhan *et al.*, 1980), and recent *in vivo* studies with mast cell-deficient mice have demonstrated a role for mast cells in tumour angiogenesis (Starkey *et al.*, 1988). Since continued tumour growth is dependent upon the ingrowth and supply of new vasculature (Kessler *et al.*, 1976) it is possible that the inhibition of tumour growth reported here with FPL 55618 is explained not only by the prevention of a direct stimulation of tumour cell proliferation, but also by a reduction in the extent of neovascularisation. At present the relative contribution of these two aspects to tumour growth are unknown, as indeed are other indirect mechanisms involving lymphocytes, macrophages and fibroblasts – all cells which may be 'activated' by mast cell mediators. Histamine is also a potential stimulus for tumour growth (Bartholeyns & Bouclier, 1984; Norrby, 1985), but it seems likely that several other mast cell growth factors such as granulocyte/macrophage colony stimulating factor (GM-CSF), interleukin-3 and other cytokines (Wodnar-Filipowicz *et al.*, 1989; Gordon *et al.*, 1990) may contribute to the behaviour of $MTLn_3$ cells at the tumour-host junction.

The tumour:host interface or 'invasion zone' of the rat mammary adenocarcinoma is variable with regard to the type and relative numbers of specific host cells, but mast cells are always conspicuous. Where mast cell degranulation was observed it was often associated with lysis of stromal connective tissue, possibly as a consequence of mast cell enzymes or the stimulation and activation of collagenolytic enzymes by neighbouring cells (Dabbous *et al.*, 1986*a*,*b*). Such activities, together with the production of growth factors and localised oedema would promote the disruption of homeostasis and could well favour tumour growth and invasion. The reduction in tumour growth effected by the mast cell-stabilising compound FPL 55618 supports the hypothesis that local mast cell activation at the tumour peripherpy contributes significantly to tumour growth and development.

This work was supported by USPHS Grants CA-25617, CA-44352 and the Cancer Research Campaign, UK. Part of the work was also supported by a Yamagiwa-Yoshida Memorial International Cancer Study Grant to DEW.

### References

AZIZKHAN, R.G., AZIZKHAN, J.C., ZETTER, B.R. & FOLKMAN, J. (1980). Mast cell heparin stimulates migration of capillary endothelial cells *in vitro*. *J. Exp. Med.*, **152**, 931.

BAEZA, M.L., REDDIGARI, S., HAAK-FREDNSCHO, M. & KAPLAN, A.P. (1989). Purification and further characterization of human mononuclear cell histamine-releasing factors. *J. Clin. Invest.*, **83**, 1204.

BARTHOLEYNS, J. & BOUCLIER, M. (1984). Involvement of histamine in growth of mouse and rat tumours: antitumoural properties of monofluoromethylhistidine. *Cancer Res.*, **44**, 639.

BARTHOLOMAEUS, W., BRAY, A.E., PAPADIMITRIOU, J.M. & KEAST, D. (1974). Immune response to a transplantable malignant melanoma in mice. *J. Natl Cancer Inst.*, **53**, 1065.

BARTHOLOMAEUS, W.N. & KEAST, D. (1972). Reaginic antibody to tumour and alloantigens in mice. *Nature New Biol.*, **239**, 206.

BAUER, E.A., UITTO, J., WALTERS, R.C. & EISEN, A.Z. (1979). Enhanced collagenase production by fibroblasts derived from human basal cell carcinoma. *Cancer Res.*, **39**, 4594.

BISWAS, C. (1982). Tumour cell stimulation of collagenase production by fibroblasts. *Biochem. Biophys. Res. Commun.*, **109**, 1026.

BROMLEY, M. & WOOLLEY, D.E. (1984). Histopathology of the rheumatoid lesion:identification of cell types at sites of cartilage erosion. *Arthritis Rheum.*, **27**, 857.

BROOM, B.C. & ALEXANDER, P. (1975*a*). Mast cell and anaphylactic antibody responses in inbred rats to synergic fibrosarcomas. *Int. Archs. Allergy. Appl. Immun.*, **49**, 627.

BROOM, B.C. & ALEXANDER, P. (1975*b*) Rat tumour allografts evoke anaphylactic antibody responses. *Immunology*, **28**, 1033.

CAIRNS, H. (1980). New, orally effective chromone derivatives for the treatment of asthma. In *Drugs Affecting the Respiratory System*. D. Temple. (ed.). American Chem. Soc. p. 99. Washington DC.

CSABA, G., ACS, T., HORVATH, C. & MOLD, K. (1961). Mast cell and plasmacyte reaction to induced, homologous and heterogenous tumours. *Br. J. Cancer*, **15**, 327.

DABBOUS, M.Kh., EL-TORKY, M., HANEY, L., BRINKLEY, Sr.B. & SOBHY, N. (1983). Collagenase activity in rabbit carcinoma: cell source and cell interactions. *Int. J. Cancer*, **31**, 357.

DABBOUS, M.Kh., WALKER, R., HANEY, L., CARTER, L.M., NICOLSON, G.L. & WOOLLEY, D.E. (1986*a*). Mast cells and matrix degradation at sites of tumour invasion in rat mammary adenocarcinoma. *Br. J. Cancer*, **54**, 459.

DABBOUS, M.Kh., WOOLLEY, D.E., HANEY, J., CARTER, L.M. & NICOLSON, G.L. (1986*b*). Host mediated effectors of tumour invasion: role of mast cells in matrix degradation. *Clin. Exp. Metast.*, **4**, 141.

EHRLICH, P. (1879). Beitrage zur Kenntniss der granulierten Bindegewebzellen und der eosinophilen leucocyten. *Arch. Anat. Physiol.*, **3**, 166.

FIDLER, I.J. & HART, I.R. (1982). Biologic diversity in metastatic neoplasms: origins and implications. *Science*, **217**, 889.

GORDON, J.R., BURD, P.R. & GALLI, S.J. (1990). Mast cells as a source of multifunctional cytokines. *Immunol. Today*, **11**, 458.

GRAHAM, R.M. & GRAHAM, J.B. (1966). Mast cells and cancer of the cervix. *Surg. Gynecol. Obstet.*, **123**, 3.

GRUBER, B.L., MARCHASE, M.J., SUZUKI, K., SCHWARTZ, L.B., OKADA, Y., NAGASE, H. & RAMAMURTHY, N.S. (1989). Synovial procollagenase activation by human mast cell tryptase. Dependence upon matrix metalloproteinase 3 activation. *J. Clin. Invest.*, **84**, 1657.

HARTVEIT, F. (1981). Mast cells and metachromasia in human breast cancer: their occurrence, significance and consequences: a preliminary report. *J. Path.*, **134**, 7.

HARTVEIT, F., THORESEN, S., TANGEN, M. & MAARTMANN-MOE, H. (1984). Mast cell changes and tumour dissemination in human breast carcinoma. *Invasion & Metastasis*, **4**, 146.

IONOV, I.D. (1989). Mast cell-basophil system in tumour growth. *Neurofibromatosis*, **2**, 204.

KESSLER, D.A., LANGER, R.S., PLESS, N.A. & FOLKMAN, J. (1976). Mast cells and tumour angiogenesis. *J. Cancer*, **18**, 703.

LEWIS, R.A. & AUSTEN, K.F. (1981). Mediation of local homeostasis and inflammation by leukotrienes and other mast cell compounds. *Nature*, **293**, 103.

LIOTTA, L.A. (1984). Warner-Lambart Parke-Davis Award. *Amer. J. Pathol.*, **117**, 339.

LIOTTA, L.A., THORGEIRSSON, U.P. & GARBISA, S. (1982). Role of collagenases in tumour cell invasion. *Cancer Metastasis Rev.*, **1**, 277.

NERI, A. & NICOLSON, G.L. (1981). Phenotypic drift of metastatic and cell surface properties of mammary adenocarcinoma cell clones during growth *in vitro*. *Int. J. Cancer*, **28**, 731.

NERI, A., WELCH, D.R., KAWAGUCHI, T. & NICOLSON, G.L. (1982). Development and biologic properties of malignant cell sublines and clones of a spontaneously metastasizing rat mammary adenocarcinoma. *J. Natl Cancer. Inst.*, **68**, 507.

NICOLSON, G.L. (1988). Organ specificity of tumour metastasis: role of preferential adhesion, invasion and growth of malignant cells at specific secondary sites. *Cancer Metastasis Rev.*, **7**, 143.

NORRBY, K. (1985). Evidence of mast cell histamine being mitogenic in intact tissue. *Agents Actions*, **16**, 287.

NORRBY, K. & ENESTROM, S. (1984). Cellular and extracellular changes following mast-cell secretion in a vascular rat mesentary. *Cell Tissue Res.*, **235**, 339.

NORDLUND, J.J. & ASKENASE, P.W. (1983). The effect of histamine, antihistamine, and a mast cell stabilizer on the growth of Cloudman melanoma cells in DBA/2 mice. *J. Invest. Dermatol.*, **81**, 28.

PARWARESCH, M.R., HORNY, H.-P. & LENNART, K. (1985). Tissue mast cells in health and disease. *Path. Res. Pract.*, **179**, 439.

ROCHE, W.R. (1985). Mast cells and tumours: the specific enhancement of tumor proliferation *in vitro*. *Am. J. Path.*, **119**, 57.

ROCHE, W.R. (1986). The nature and significance of tumour-associated mast cells. *J. Pathol.*, **148**, 175.

SCHWARTZ, L.B. (1989). Heterogeneity of mast cells in humans: In: *Mast Cell and Basophil Differentiation and Function in Health and Disease*. Galli, S.J. & Austen, K.F. (eds). p. 93. Raven Press: NY.

SCOTT, K.G. (1963). The mast cell, its amines, and tumor growth in rodents and man. *Ann. N.Y. Acad. Sci.*, **103**, 285.

SELYE, M. (1965). The mast cells, *Butterworths*, Washington.

SERAFIN, W.E. & AUSTEN, K.F. (1987). Mediators of immediate hypersensitivity reactions. *New Eng. J. Med.*, **317**, 30.

SMYTH, C.J. & GUM, O.B. (1958). Mast cells in connective tissue diseases. *Arthritis Rheum.*, **1**, 178.

STARKEY, J.R., CROWE, P.K. & TAUBENBURGER, S. (1988). Mast cell-deficient $W/W^v$ mice exhibit a decreased rate of tumour angiogenesis. *Int. J. Cancer*, **42**, 48.

SUSCHITZKY, J.L. & SHEARD, P. (1984). The search for antiallergic drugs for the treatment of asthma – problems in finding a successor to sodium cromoglycate. *Prog. Med. Chem.*, **21**, 1.

SWEENEY, W.T. & SEIBEL, H.R. (1973). Histamine release from peritoneal mast cells of tumour susceptible rats following periods of tumor growth and sensitization with tumor antigen and *B. pertussis*. *Int. Archs. Allergy Appl. Immunol.*, **45**, 789.

WODNAR-FILIPOWICZ, A., HEUSSER, C.H. & MORONI, C. (1989). Production of the haemopoietic growth factors GM-CSF and interleukin-3 by mast cells in response to IgE-receptor-mediated activation. *Nature*, **339**, 150.

WOOLLEY, D.E. (1982). Collagenase immunolocalisation studies of human tumours. In: *Tumour Invasion and Metastasis*. Liotta, L.A. & Hart, I.R. (eds) p. 391. Martinus Nijhoff: The Hague.

WOOLLEY, D.E. (1984). Collagenolytic mechanisms in tumor cell invasion. *Cancer Metastasis Rev.*, **3**, 61.

YAMADA, K.M., AKIYAMA, S.K., HASEGAWA, T. & 5 others (1985). Recent advances in research on fibronectin and other cell attachment proteins. *J. Cell. Biochem.*, **28**, 79.

YOFFE, J.R., TAYLOR, D.J. & WOOLLEY, D.E. (1984). Mast cell products stimulate collagenase and prostaglandin E production by cultures of adherent rheumatoid synovial cells. *Biochem. Biophys. Res. Commun.*, **122**, 270.